# Target size, self-efficacy, and stress as determinants of precision in a marksmanship task

**DOI:** 10.3389/fpsyg.2025.1710147

**Published:** 2026-01-06

**Authors:** Fabio Ibrahim, Kevin Wittig, Philipp Yorck Herzberg

**Affiliations:** Department of Personality Psychology and Psychological Assessment, Helmut-Schmidt-University, Hamburg, Germany

**Keywords:** heart rate variability, marksmanship, military performance, self-efficacy, shooting, stress, target size

## Abstract

Marksmanship is a critical skill for law enforcement and military personnel, serving as a last resort in life-threatening situations to protect civilians, teammates, and oneself. While many studies have examined factors influencing shooting precision, the role of target size as a peripheral feature remains underexplored. This study investigated the effects of target size, shooting self-efficacy, subjective stress, shooting experience, physiological stress, and stress state variability on precision. A total of *n* = 140 student officers (74% male; *M* = 23.5 years) completed two live-fire tasks in a shooting simulator, firing ten rounds each at a small (12 cm) and a large (30 × 25 cm) target area with an identical aim point. Measures included emotional stress reactions, self-efficacy, shooting experience, and heart rate variability (RMSSD). Precision was indexed via mean distance to center and shot group radius. Smaller targets significantly enhanced precision (d = 0.36) independent of subjective stress. Self-efficacy predicted performance (*r* = 0.39) and was negatively associated with subjective stress (*r* = −0.30) and stress variability (*r* = −0.18). Mediation analysis showed that subjective stress partially explained the link between self-efficacy and precision (17.7%). RMSSD was unrelated to precision, whereas stress variability correlated positively with performance instability (*r* = 0.21). These findings suggest that smaller target areas act as peripheral cues that support perceptual-motor alignment during the limb–target control phase. Moreover, psychological attributes such as shooting self-efficacy contribute to performance both directly and via stress reduction. The results identify modifiable factors in shooting precision that can be systematically addressed in marksmanship training.

## 1 Introduction

It was the afternoon of May 3, 2015, in Garland, Texas. An anti-Islam event held at the Curtis Culwell Center with approximately 150 attendees was monitored by police officer Gregory Stevens. Stevens reported a sense of unease at the event, stating: “*This had a lot of potential for bad things happening*” (CBS News, 2019). Without prior warning, two individuals wearing body armor and armed with assault rifles exited a vehicle and approached the event with the intention of causing mass casualties. Officer Stevens, armed only with his standard sidearm, immediately engaged the assailants. Despite being outgunned, he neutralized both attackers with remarkable precision from a distance of over 30 feet within 15 s. He later commented: “*This whole event probably did not take no more than 10 and probably 15 s. I’m a pretty good shooter. I’m not a great shooter. My training kicked in*” (CBS News, 2019).

This case highlights the reality that professionals in high-risk occupations such as law enforcement or military service must constantly anticipate unpredictable threats. The use of a firearm is regarded as a last resort to protect civilians (Taylor, 2019), teammates, and one’s own life (Anderson et al., 2002). Therefore, the ability to shoot with precision under pressure is a critical skill for these professions. However, not all officers or soldiers possess such capabilities. For instance, the New York Police Department reported a hit rate of only 30% in incidents without return fire and 18% when under attack (Rostker, 2008). Marksmanship is thus a fundamental competence in high-risk professions, and the investigation of factors influencing shooting performance is a central concern of operational psychology.

## 2 Theoretical background

Shooting with a handgun is a task that demands high levels of precision, emotional stability, and stress adaptability (Lu et al., 2022). A systematic review by Spancken et al. (2021) identified factors influencing shooting accuracy, which can be grouped into three categories: technical-coordinative variables (e.g., weapon stability, trigger control, body posture), physiological variables (e.g., heart rate), and psychological variables (e.g., anxiety, subjective stress experience). Shooting is therefore a complex psychomotor behavior influenced by multiple interacting factors.

According to the model of speed-accuracy in goal-directed reaching by Elliott et al. (2017), goal-directed actions such as reaching, pointing, or shooting consist of two distinct phases aimed at optimizing speed and accuracy. In the *impulse control phase* (predictive feedforward control), a preplanned movement is executed rapidly with minimal sensory correction to bring the limb close to the target. In the *limb-target control phase* (sensory feedback control), the limb is then adjusted using visual and proprioceptive information to align precisely with the target. The limb-target control phase requires high concentration and the integration of sensory feedback to accurately determine the spatial relationship between the limb and the target. In addition to aligning the weapon, the shooter must manage the trigger and account for external stressors.

### 2.1 External factors: time constraints, threat, task complexity

According to the speed-accuracy model, there is a tradeoff between speed and precision, especially evident in the limb-target control phase. Limited time reduces opportunities for correction during the limb-target alignment, thus decreasing precision (Zhao et al., 2024). Moreover, time pressure increases psychological stress, which in turn impairs performance. Arble et al. (2019) showed that various cognitive demands such as perceived threat, task complexity, or external constraints resulted in increased physiological arousal, perceived pressure, and state anxiety in urban police officers. These external stressors influence performance by altering gaze behavior, reducing response accuracy, delaying psychomotor execution, and ultimately diminishing shooting precision (Nieuwenhuys et al., 2012; Nibbeling et al., 2014).

Stress management techniques, such as tactical breathing, have been shown to enhance marksmanship under pressure (Ibrahim et al., 2024a). These findings are supported by a meta-analysis by Cooper et al. (2024), which reported that perceived pressure decreased shooting accuracy by 14.8% and slightly increased the likelihood of decision errors. In a study by Nieuwenhuys and Oudejans (2010), police officers participated in a force-on-force (FoF) scenario involving active armed opponents using colored soap cartridges. The scenario significantly increased stress and reduced shooting accuracy; however, this performance decline was mitigated by prior exposure to FoF training. Cooper et al. (2024) concluded from their meta-analysis that early exposure to pressure-based training, such as FoF scenarios, improved performance by 10.6% compared to traditional training.

In line with the attentional control theory (Eysenck et al., 2007), heightened anxiety, such as when facing an armed opponent, reduces goal-directed top-down processing and increases stimulus-driven bottom-up processing to detect environmental threats. Officers exposed to more anxiety-inducing training conditions exhibited faster shooting, less direct opponent fixation, and increased blink rates. Nieuwenhuys and Oudejans (2010) concluded that under stress, goal-directed attention decreases while stimulus-driven attention increases, reflected in visual fixations on the weapon or the opponent’s head. Also, in high-stakes tactical scenarios such as close quarters battle (CQB), significant differences in gaze behavior and visual information processing have been observed between novices and expert units (Ibrahim et al., 2024a). Prolonged visual fixation on the target area is generally associated with higher shooting precision (Vickers, 2007), likely because it facilitates accurate sensory feedback during the limb–target control phase.

### 2.2 Psychological dispositions: confidence, anxiety, and experience

According to Spancken et al. (2021), psychological dispositions, alongside technical and physiological factors, have a significant impact on shooting performance. Mat Salleh et al. (2023) found a strong positive correlation between self-confidence and shooting accuracy in archers. In military samples using firearms, self-efficacy was moderately associated with performance (Anglin et al., 2017). Similarly, decision-related action orientation, defined as a tendency to act with initiative and focus, predicted marksmanship under high pressure (Landman et al., 2016). Emotional regulation traits such as anxiety variability (Kayihan et al., 2013) and trait anxiety (Simas et al., 2022) were predictors of shooting performance. Studies on Big Five personality traits indicated that openness was negatively associated with marksmanship (Buskerud et al., 2022), and extraversion was inversely related to performance in CQB tasks (Ibrahim et al., 2024b). Neuroticism, as a personality dimension linked to emotional instability and negative affect, was also found to be a negative predictor of shooting precision in police students (Löfgren and Hansson, 2022).

Experience also plays a critical role. A meta-analysis by Cooper et al. (2024) found that years of service mitigated the negative effects of stress on shooting performance. In competitive air pistol shooting, less experienced athletes showed higher anxiety and lower precision (Zhao et al., 2024). In contrast, self-reported firearm capability in a military sample did not predict shooting performance, likely due to individual reference biases in self-assessment (Biggs et al., 2024).

Beyond the established speed–accuracy relationship, the broader literature on stress and performance highlights that elevated arousal does not uniformly impair motor execution. According to the Yerkes–Dodson law, performance follows an inverted-U function in which moderate levels of arousal may facilitate, rather than hinder, task execution. This pattern is evident in police and military training studies. Giessing et al. (2019) found that novice police recruits exhibited markedly higher perceived anxiety and substantially reduced vagal control (approximately 50% lower HRV) during their first exposure to a high-stress reality-based shooting scenario, yet their shooting accuracy remained comparable to low-stress conditions. Similarly, recent evidence shows that stress induced by unfamiliarity or novel task demands may not degrade performance and can even enhance attentional engagement. These findings underscore that stress effects on marksmanship are context dependent and may vary as a function of arousal intensity, task novelty, and individual differences in stress reactivity.

### 2.3 Physiological variables: stress, heart rate, and heart rate variability

According to appraisal theory, emotions such as fear arise not directly from physiological arousal but from the cognitive evaluation of arousal within a situational context (Reisenzein, 2000). In the context of shooting, this includes perceived threat, shooting self-efficacy, and target characteristics. Physiological responses such as heart rate (HR) and heart rate variability (HRV) have emerged as reliable indicators of distress during performance under pressure. For example, elite archers exhibited lower mean heart rates and higher HRV than intermediate shooters (Mat Salleh et al., 2023).

Thompson et al. (2015) found that smaller reductions in HRV under stress were associated with higher shooting accuracy, suggesting that HRV-derived sympathetic response could serve as a biomarker for performance. Zhao et al. (2024) observed a relationship between pre-competition anxiety, inter-competition HRV, and shooting precision, with elite athletes showing higher HRV during performance.

In addition to these findings, recent research suggests that HRV may influence performance through mechanisms extending beyond stress regulation. Spangler et al. (2018) demonstrated that higher HRV protected against negative operand conditioning and improved friend–foe decision-making under pressure, indicating that autonomic flexibility contributes to adaptive cognitive control in tactical contexts. Related evidence shows that age and physical activity also predict future shooting performance in reaction-time based transition tasks (Buga et al., 2025), further underscoring that physiological conditioning and experiential factors jointly shape performance under stress. These results support the conceptualization of HRV not only as a stress marker but also as a potential index of decision-making resilience in high-stakes environments.

In summary, multiple factors affect shooting performance. Spancken et al. (2021) categorized these into technical, physiological, and psychological domains. The physiological domain primarily includes heart rate (HR) and heart rate variability (HRV), with lower physiological arousal being associated with better shooting performance (Zhao et al., 2024; Mat Salleh et al., 2023). In the psychological domain, key influencing factors include trait anxiety and anxiety variability (Kayihan et al., 2013), shooting self-efficacy (Anglin et al., 2017), and neuroticism (Löfgren and Hansson, 2022).

The technical domain is particularly well explained by the model of speed-accuracy in goal-directed reaching (Elliott et al., 2017), especially the limb-target control phase, which relies heavily on visual and proprioceptive feedback to align the weapon. In this phase, the spatial perception of the limb and the target becomes central.

To our knowledge, one important parameter in the limb-target control phase has not yet been systematically examined in this context: target size. According to Fitts’ law (Fitts, 1954), a fundamental speed and accuracy tradeoff exists, such that more precise aiming typically requires more time during the limb-target control phase of motor execution. While target size varies substantially in real-world scenarios, such as the incident involving Officer Gregory Stevens at a distance of 30 feet, its specific impact on marksmanship remains largely unexamined. Despite the central aim of hitting the ideal point, comparable to the theoretical optimum or centerline, remaining constant across targets, it is unclear whether variations in the target area affect shooting performance. This raises the question of whether perceptual differences induced by varying target sizes, despite an identical point of aim, affect the precision of goal directed firearm use.

Evidence from human-computer interaction suggests that smaller target areas on trackpad interfaces result in greater motor precision, likely due to enhanced attentional focus and increased movement impedance (Huysmans et al., 2012). It remains unclear whether such effects translate to shooting under realistic conditions. Moreover, according to the findings of Nieuwenhuys and Oudejans (2010), increased stress leads to stronger stimulus-driven attention. This raises the question of whether stress amplifies the influence of the target area on shooting precision by enhancing bottom-up processing during the limb–target control phase.

### 2.4 The current study

Although motor skills, attentional control, and stress management have been extensively studied in marksmanship research, the potential role of the visual target medium itself remains largely unexplored. Altering the surrounding visual context of a fixed aiming point could influence attentional focus, perceptual-motor coordination, and performance consistency under stress. Therefore, the present study examines, the influence of the target area on shooting precision in relation to shooting self-efficacy and stress during live fire in a shooting simulator. Specifically, we investigate whether targets of different sizes, despite an identical point of aim, affect shooting accuracy; whether stress impairs performance and amplifies bottom-up processing, as indicated by precision differences across target sizes; and whether stress mediates the relationship between shooting self-concept and performance.

We hypothesize that

H1a: A smaller target area increases shooting precision.

H1b: A higher level of subjective stress (high negative–positive emotions balance score) are associated with lower shooting precision.

H1c: A stronger physiological stress response (lower HRV) is associated with lower shooting precision.

H1d: The shooting self-efficacy is positively associated with shooting precision.

H1e: Shooting experience is positively associated with shooting precision.

H1f: The shooting self-efficacy is negatively associated with subjective stress.

H2a: Subjective stress moderates the effect of target size on shooting precision, such that the positive effect of a smaller target area is attenuated when subjective stress is higher.

H2b: Subjective stress mediates the effect of shooting self-efficacy on shooting precision.

H3a: Greater stress state variability between shootings is associated with a larger precision difference between large and small targets.

H3b: Shooting self-efficacy predicts a lower stress state variability between shootings.

## 3 Methods

### 3.1 Procedure

The experiment was designed as a mixed-subject design, with target size serving as a within subject factor, and demographic variables, shooting self-efficacy, and shooting experience as between-subject factor. Data collection was conducted within the German Armed Forces firing simulator, which allows for realistic shooting scenarios using compressed air weapons. A total of seven data collection sessions took place between June 18 and October 16, 2024.

**Figure 1:**
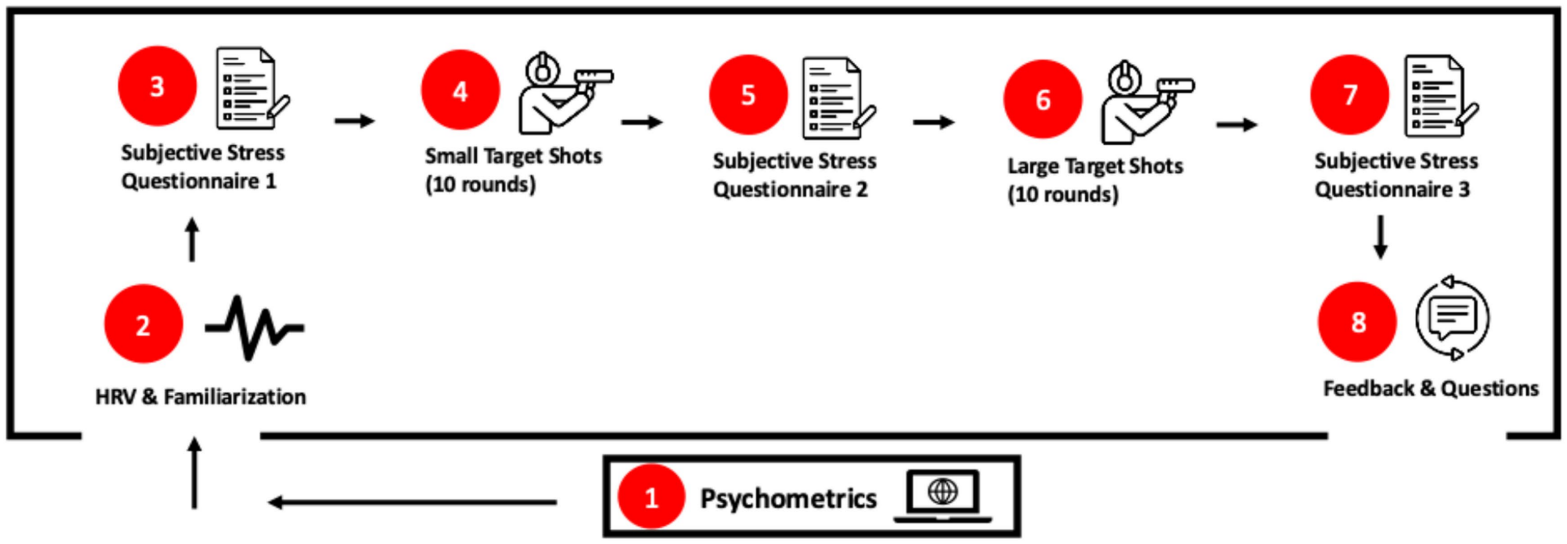
Experimental timeline of the study protocol. The figure illustrates the sequential order of all procedures conducted within a single session: (1) baseline psychometric assessment; (2) HRV setup and brief familiarization with the simulator; (3) subjective stress questionnaire 1; (4) small-target shooting block (10 rounds); (5) subjective stress questionnaire 2; (6) large-target shooting block (10 rounds); (7) subjective stress questionnaire 3; and (8) instructor feedback on shooting performance.

**Figure 2:**
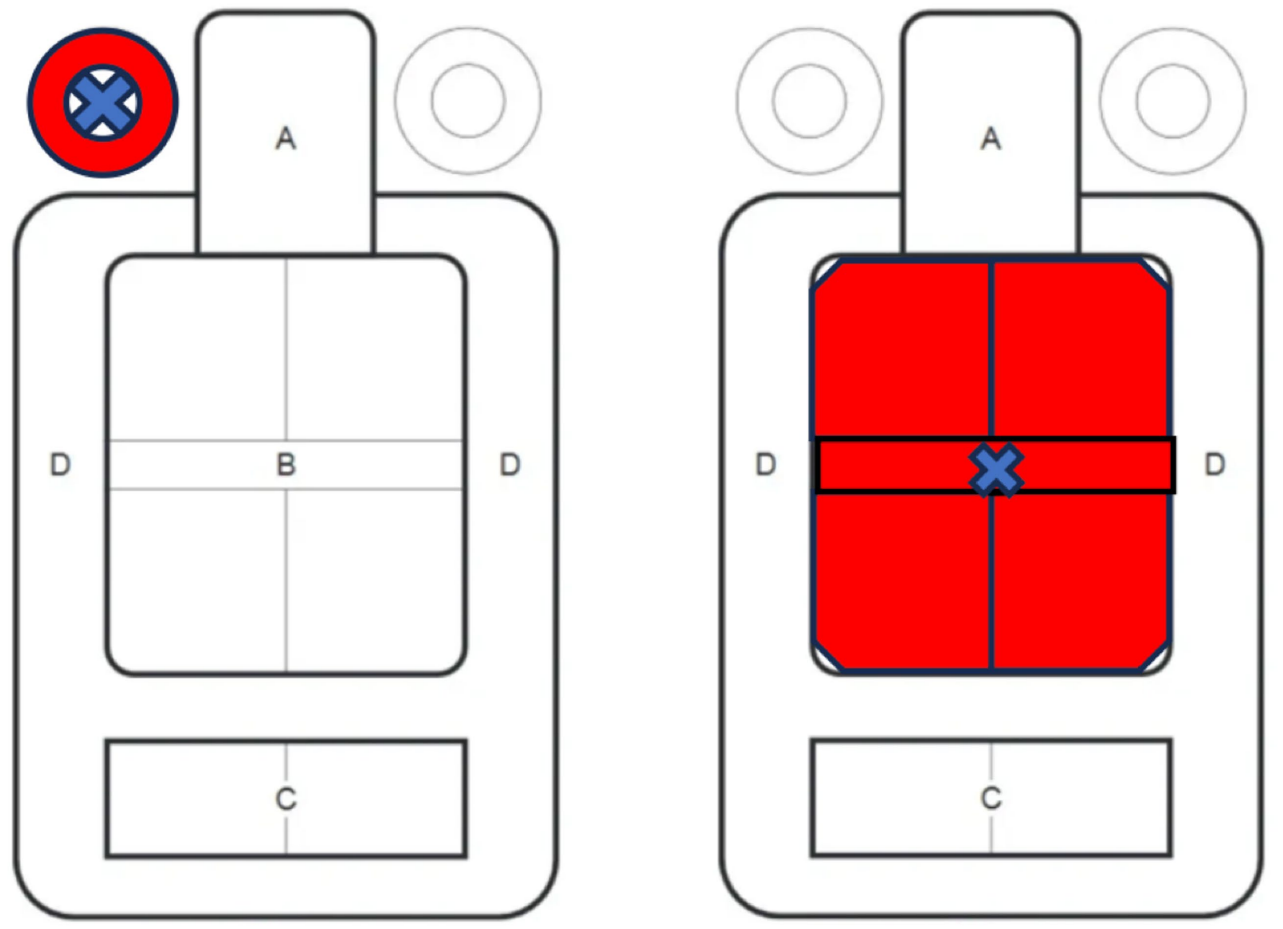
T-target stimulus used in the shooting simulator. Left: Small target area highlighted in red (diameter = 12 cm) with a blue aiming point; Right: Large target area highlighted in red (30 cm × 25 cm) with a blue aiming point; The aim point was identical for both conditions.

To improve transparency, Figure 1 provides an overview of the complete experimental timeline. All procedures were conducted within a single session:

1. Participants were informed about the study aims and data protection protocols. They then generated a personal pseudonym, allowing for anonymized data linkage. Subsequently, they completed an online questionnaire on-site using SoSciSurvey version 3.2.21. The questionnaire assessed sociodemographic data, shooting experience, shooting self-efficacy, and subjective stress prior to live fire execution. The completion took approximately 7 min.
2. Next, the H7 Heart Rate Sensor (Polar Electro, 2014) was attached, and its functionality was checked. Because all participants were active-duty soldiers with prior live-fire P8 qualification, only a short familiarization period (1–2 min) with the simulator interface and recoil simulation was required. No practice shots were fired. Moreover, all participants had previously received basic instruction on the AGSHP simulator as part of their standard military training, ensuring pre-existing familiarity with the system’s operating principles. Although multi-session VR learning protocols (Chung et al., 2009) can require extensive training time, an extended familiarization phase was not necessary in the present study because participants already possessed both real-weapon experience and prior exposure to the simulator environment.
3. After familiarization, participants completed the first subjective stress questionnaire.
4. Participants then were instructed to fire ten rounds at the small target, defined as the left ring next to the T-target. The instructions emphasized hitting the blue cross, i.e., the designated center point of aim (Figure 2). The small target had a diameter of 12 cm, and the shooting distance was 15 meters.
5. After shooting, participants completed the second subjective stress questionnaire based on their experience of shooting at the small target.
6. Next, participants were then instructed to fire ten rounds at the large target, again with emphasis on aiming at the blue cross at the center. The shooting distance remained 15 meters, while the dimensions of the large target were 30 by 35 cm.
7. Following this second shooting task, participants once again completed the subjective stress questionnaire, this time in reference to the large target.
8. At the end of the session, participants received individualized feedback from the instructors regarding their shot grouping and recommendations for improving shooting performance.

### 3.2 Simulator

The study was conducted using the *Ausbildungsgerät Schießsimulator Handfeuerwaffen/Panzerabwehrhandwaffen* (AGSHP Evolution; Thales Defence Deutschland GmbH), the standard shooting simulator of the German Armed Forces. The AGSHP system employs an infrared-based optical tracking unit that records weapon alignment and trigger events at high temporal resolution. The Heckler & Koch P8 pistol used in the simulator is a real P8 service weapon equipped with a modified barrel, an integrated infrared transmitter and a compressed-air recoil module. This provides realistic recoil dynamics without discharging actual projectiles and preserves the weapon’s original weight, dimensions, and trigger characteristics. Comparable AGSHP configurations have been used in military and police training and have demonstrated high face validity and stable performance metrics across repeated sessions (e.g., Biggs et al., 2024; Ibrahim et al., 2024c).

The targets presented in the simulator were standardized Bundeswehr “T-Scheibe” (T-target) silhouettes. This target type is routinely used in German Armed Forces qualification courses due to its high recognizability and its suitability for evaluating precision at fixed distances. The use of the T-target ensured comparability with operational training standards and allowed for controlled manipulation of the peripheral target area while keeping the aim point constant. The target configuration and the 15-meter shooting distance fall within the permitted training range for the near and next-to-near engagement zone (5 to 25 meters; Jamro et al., 2022). According to the certified shooting instructors, a distance of 15 meters represents an appropriately challenging, yet not overwhelming, marksmanship task for trained soldiers.

### 3.3 Participants

The study was conducted at the Helmut-Schmidt-University/University of the German Armed Forces in Hamburg. Participation was voluntary and compensated through course credit. The recruitment took place via the university’s internal bulletin mailing list. Eligibility criteria included the absence of physical or psychological impairments and active-duty status as a member of the German Armed Forces. The initial sample consisted of *n* = 153 participants. Four cases were excluded due to missing shooting performance data and nine cases due to incomplete HRV recordings. The resulting final sample comprised *n* = 140 complete data sets, including 103 men (74%) and 37 women (26%), with a mean age of 23.5 years ([19–35], *SD* = 3.41, *Md* = 23.00).

All participants were adequately trained in marksmanship according to German Armed Forces standards. Each participant had completed the mandatory P8 pistol qualification during basic training, and all had undergone advanced marksmanship instruction as part of their officer school curriculum. In the year of data collection, participants reported an average of 5.91 shooting training days (SD = 8.24; range = 0–45), indicating substantial variation but generally consistent ongoing exposure to live-fire practice.

All participants provided informed consent in accordance with the Declaration of Helsinki. The study protocol was reviewed and approved by the Internal Ethics Committee of the Helmut Schmidt University (reference number: 2024_019). The study was preregistered at the Open Science Framework,^1^ and the dataset is publicly available at https://osf.io/w72pn/.

### 3.4 Physiological and psychological measurements

#### 3.4.1 Emotional stress reaction questionnaire (ESRQ)

The German-language ESRQ (Larsson, 2011) comprises 14 items, each rated on a four-point Likert scale ranging from 1 (*does not apply at all*) to 4 (*fully applies*). The scale assesses emotional responses to stressful situations within 60 s and is specifically designed for use in operational psychology. It consists of 14 emotion words (e.g., “heated”) that reflect four appraisal categories: Irrelevant, Benign-positive, Challenge, and Threat, harm or loss. The negative–positive emotions balance score is calculated by subtracting the positive emotions (Benign-positive; Challenge) from the negative ones (Irrelevant; Threat, harm or loss). The scale shows good to excellent internal consistency (*α* = 0.74–0.90; Larsson, 2011).

#### 3.4.2 Shooting self-efficacy

To assess shooting self-efficacy, a German-language instrument specifically designed for the use of the P8 handgun was developed. The scale comprises eight items rated on a four-point Likert scale ranging from 1 (*does not apply at all*) to 4 (*fully applies*). It was constructed in line with the self-efficacy concept, defined as confidence in one’s ability to successfully perform specific tasks (Bandura, 1977). The items cover the domains of safety and competence (“I can handle the P8 safely”); training (“I have received sufficient training with the P8”); ease (“Shooting with the P8 is easy for me”; “I look forward to shooting exercises”); aptitude (“I have a talent for handling the P8”; “I am accurate”); comparison (“I shoot better than others with the P8”); and relevance (e.g., “It is important to me to be able to shoot well with the P8”). In the present sample, the scale demonstrated very good reliability (α = 0.85).

#### 3.4.3 Heart rate variability

Heart rate variability (HRV) refers to the variation in the time interval between heartbeats and serves as an indicator of autonomic nervous system activity, with higher HRV reflecting greater vagal tone (Hourani et al., 2020). Vagal tone is considered an indicator of stress and stress vulnerability (Porges, 1995) and is frequently used as a non-invasive physiological marker of stress responses in military populations (Lewis et al., 2015). In this study, the H7 Heart Rate Sensor and the Polar V800 sports watch (Polar Electro, 2014) were used. The H7 Heart Rate Sensor has been shown to be a valid instrument for measuring HR and HRV (Chow and Yang, 2020). Data were analyzed using Kubios HRV Lite software (Tarvainen et al., 2014) to calculate the Root Mean Square of Successive Differences (RMSSD). RMSSD was selected as the HRV parameter because it is less susceptible to fluctuations and is considered a more valid indicator for short-term measurements (Laborde et al., 2017).

#### 3.4.4 Shooting experience

Deliberate practice, that is, the purposeful investment of time in training, is considered an important predictor of skill development in competitive sports (Macnamara et al., 2016). Accordingly, participants in this study were asked to report the number of days they had engaged in shooting practice during the last 12 months as an indicator of shooting experience.

#### 3.4.5 Shooting performance

Two measures were used to quantify shooting precision. The mean distance to center (MDC) represents the average distance of each hit from the target’s center and serves as an indicator of accuracy. Second, the hit group size (radius) was measured, reflecting the consistency of performance (Buskerud et al., 2022). For ease of interpretation, both performance indicators were inverted so that higher values uniformly reflected higher shooting precision across all analyses. In their original form, lower mean distance to center (MDC) and smaller shot group radius indicate better performance. However, because all psychological predictors in this study (e.g., self-efficacy, experience) were positively valenced constructs, inverting MDC and radius ensured that higher values consistently represented superior performance across variables.

### 3.5 Analytic technique

Data analyses were conducted using R (Team, 2022) with the packages lavaan (Rosseel et al., 2017) and lme4 (Bates et al., 2015). Only complete datasets were included. Outliers were examined using Mahalanobi’s distance, and individuals with a d-squared value *p* < 0.001 were examined. Paired-samples t-tests were conducted to examine the simple effect of target size on shooting precision, and Pearson correlations were calculated to assess bivariate associations. Given the mixed design, with target size manipulated within participants and other predictors (e.g., shooting self-efficacy, shooting experience) varying between participants, linear mixed-effects models were used to account for both within- and between-subject variability. Mediation analyses were performed using mixed-effects models and bootstrapping (Preacher and Hayes, 2004), and moderation analyses with lavaan. The statistical power was high for detecting medium-sized effects, as correlations of *r* ≥ 0.27 could be detected with a 5% type I error rate and 90% power.

## 4 Results

### 4.1 Descriptive statistics

A multivariate outlier analysis (Mahalanobis distance, *p* < 0.001) identified four potential outliers. These cases were retained due to plausible response patterns upon inspection, resulting in a final sample of *n* = 140 participants. Descriptive statistics for the total sample are presented in Table 1.

**Table 1:** Sample demographics.

Category	Variable	Male	Female	Cohen’s d
Demog.	Male in %	103 (74%)	37 (26%)	
	Age (years)	23.63 (3.53)	23.03 (3.09)	0.18
	Shooting experience (days)	6.01 (8.18)	5.62 (8.51)	0.05
	Shooting self-efficacy	3.04 (0.51)	2.61 (0.58)	0.81
Emotional stress reaction	ESRQ			
	M1 Positive	20.05 (3.19)	18.19 (3.47)	0.57
	M1 Negative	9.94 (2.75)	10.43 (3.38)	−0.17
	M1 Positive–Negative	10.11 (4.93)	7.76 (5.79)	0.45
	M2 Positive	21.03 (3.96)	19.41 (3.94)	0.41
	M2 Negative	10.17 (2.58)	11.38 (3.71)	−0.41
	M2 Positive–Negative	10.85 (5.28)	8.03 (6.59)	0.50
	M3 Positive	21.44 (3.80)	20.30 (3.79)	0.30
	M3 Negative	9.97 (2.66)	10.0 (3.50)	−0.01
	M3 Positive–Negative	11.47 (5.36)	10.30 (6.52)	0.21
HRV	RMSSD			
	M2 RMSSD (ms)	25.91 (15.22)	26.32 (19.65)	−0.02
	M3 RMSSD (ms)	29.60 (17.73)	28.68 (20.10)	0.05
	M3 - M2 Difference RMSSD (ms)	3.69 (11.93)	2.37 (14.48)	0.10
Shooting	M2 MDC (mm)	6.20 (2.21)	7.11 (2.57)	−0.39
	M2 Radius (mm)	17.05 (5.39)	19.13 (6.39)	−0.37
	M3 MDC (mm)	7.06 (2.24)	8.02 (2.99)	−0.39
	M3 Radius (mm)	21.64 (6.98)	24.57 (9.06)	−0.39
	M3 - M2 Difference MDC (mm)	0.86 (2.04)	0.91 (1.81)	−0.03
	M3 - M2 Difference Radius (mm)	4.59 (5.96)	5.44 (5.41)	−0.15

Standard deviation in brackets; the percentages have been rounded to whole numbers; Cohen’s d as effect size for measuring differences between genders; M1 = measurement pre shooting; M2 = small target; M3 = Large target; ESRQ = Emotional stress reaction questionnaire; RMSSD = Root Mean Square of Successive Differences (HRV); MDC = Mean distance to center; Radius = hit group radius.

### 4.2 Effect of target size on shooting precision

**Figure 3:**
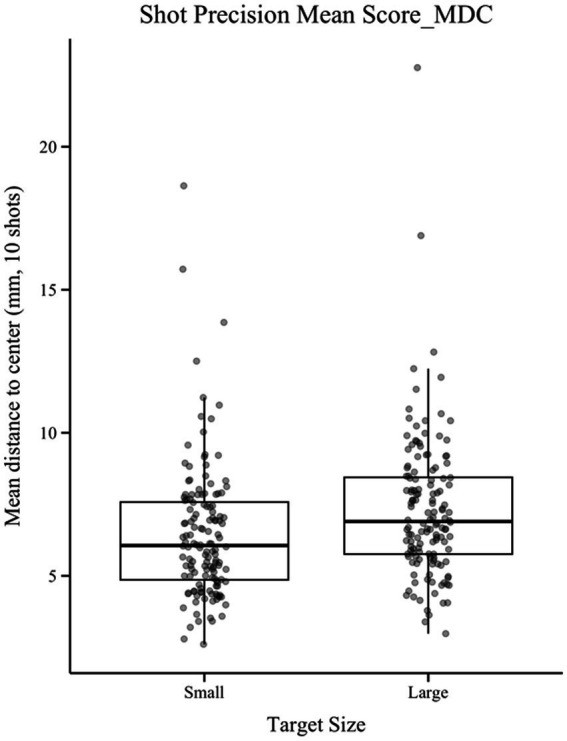
Comparison of shooting precision between the small and large target conditions; The ordinate represents mean distance to center (MDC, in mm); lower values indicate higher precision; Boxplots display median (center line), interquartile range (box).

Both shooting precision indicators were inverted for interpretability, so that higher scores indicate higher precision. Lower values of the original mean distance to center (MDC) corresponded to better shooting performance. A paired-samples *t*-test showed a significant effect of target size on MDC, *t*(139) = 5.00, *p* < 0.001, *d* = 0.36 (Figure 3), with smaller targets yielding higher precision, supporting H1a.

### 4.3 Correlational analyses

Subjective stress, measured via the negative–positive emotion balance score prior to shooting, was negatively correlated with shooting precision for both the small target (inverted MDC, *r* = −0.20, *p* = 0.020) and the large target (*r* = −0.33, *p* < 0.001), supporting H1b. Physiological stress, indexed by RMSSD, was not significantly correlated with precision for the small target (*r* = 0.09, *p* = 0.31) or the large target (*r* = 0.11, *p* = 0.20), leading to rejection of H1c. Shooting self-efficacy was positively associated with precision for the small (*r* = 0.39, *p* < 0.001) and large target (*r* = 0.27, *p* = 0.001), supporting H1d. Shooting experience was also positively associated with precision for both the small (*r* = 0.18, *p* = 0.03) and large target (*r* = 0.22, *p* = 0.01), supporting H1e. Finally, shooting self-efficacy correlated negatively with subjective stress (*r* = −0.30, *p* < 0.001), supporting H1f (Table 2).

**Table 2:** Correlations of the demographic, personality, and performance variables.

Category	Variable	1.	2.	3.	4.	5.	6.	7.	8.	9.	10.	11.	12.	13.
Demog.	1. Gender													
	2. Age	−0.08												
	3. Shooting experience	−0.02	−0.27											
	4. Shooting self-efficacy	−0.34	0.03	0.23										
ESRQ	5. M1 Negative–Positive	0.20	0.11	−0.12	−0.30									
	6. M2 Negative–Positive	0.22	0.05	−0.27	−0.46	0.36								
	7. M3 Negative–Positive	0.09	0.06	−0.21	−0.31	0.45	0.70							
HRV	8. M2 RMSSD	0.01	−0.08	0.09	0.06	−0.07	−0.09	−0.08						
	9. M3 RMSSD	−0.02	−0.01	0.05	0.12	−0.06	−0.12	−0.11	0.74					
	10. M3 - M2 Difference RMSSD	−0.05	0.09	−0.05	0.10	0.01	−0.06	−0.05	−0.23	0.49				
Shooting	11. M2 MDC	−0.17	−0.01	0.18	0.39	−0.20	−0.35	−0.28	0.09	0.10	0.03			
	12. M2 Radius	−0.16	0.01	0.20	0.34	−0.17	−0.35	−0.27	0.12	0.16	0.08	0.88		
	13. M3 MDC	−0.17	0.07	0.22	0.27	−0.02	−0.33	−0.28	0.10	0.11	0.03	0.67	0.67	
	14. M3 Radius	−0.17	0.06	0.20	0.30	−0.09	−0.37	−0.34	0.08	0.12	0.07	0.68	0.66	0.84

Cronbach’s α in brackets. Absolute correlations |r| > 0.17 / 0.22 / 0.28 correspond to *p* < 0.05 / 0.01 / 0.001. Male and female coded as 1 and 2. M1 = pre-shooting; M2 = small target; M3 = big target. ESQR = Emotional Stress Reaction Questionnaire; RMSSD = Root Mean Square of Successive Differences (HRV); MDC = inverted mean distance to center (higher values = higher precision); Radius = inverted hit group radius (higher values = smaller radius).

### 4.4 Moderation analysis

A linear mixed-effects model tested whether the effect of target size on shooting precision was moderated by within-person variations in subjective stress, controlling for between-subjects stress differences. Target size had a significant main effect (*b* = 0.89, *SE* = 0.17, *t*(276) = 5.22, *p* < 0.001), indicating higher precision for smaller targets. Between-subjects stress was negatively associated with precision (*b* = 0.15, *SE* = 0.03, *t*(276) = 4.45, *p* < 0.001). Neither the main effect of within-subject stress (*b* = 0.12, *SE* = 0.09, *t*(276) = 1.37, *p* = 0.17) nor the interaction between target size and within-subject stress (*b* = −0.19, *SE* = 0.15, *t*(276) = −1.29, *p* = 0.20) was significant. Thus, situational fluctuations in subjective stress did not significantly moderate the positive effect of smaller targets on precision, leading to the rejection of H2a.

### 4.5 Mediation analysis

**Figure 4:**
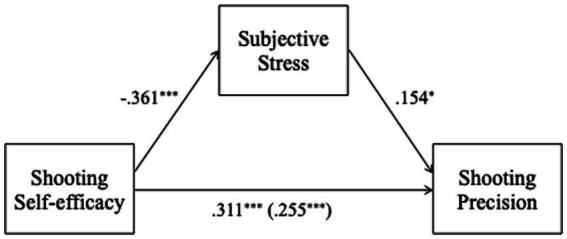
Mediation model showing the indirect effect of shooting self-efficacy on shooting precision via subjective stress. Standardized path coefficients (β) are displayed: a = effect of self-efficacy on stress; b = effect of stress on precision (controlling for self-efficacy); c′ = direct effect of self-efficacy on precision (controlling for stress); c = total effect of self-efficacy on precision. *p* < 0.05; *p* < 0.01; *p* < 0.001.

A mediation model tested whether subjective stress mediated the relationship between shooting self-efficacy and precision. Shooting self-efficacy had a significant total effect on precision (*B* = 1.37, 95% CI [0.78, 1.96], *p* < 0.001). Including subjective stress as a mediator reduced the direct effect, which remained significant (ADE = 1.13, 95% CI [0.52, 1.72], *p* < 0.001). The average causal mediation effect (ACME) was significant (ACME = 0.25, 95% CI [0.05, 0.48], *p* = 0.014), indicating that 17.7% of the total effect was explained by subjective stress. This supports a partial mediation, with higher self-efficacy predicting lower subjective stress, which in turn predicted higher precision (Figure 4). Therefore H2b could be accepted.

### 4.6 Stress state variability

Stress state variability, operationalized as the mean squared successive differences (MSSD) across the three measurement points (pre-task, post-small target, post-large target), was positively associated with the within-subject difference in precision between large and small targets (*r* = 0.21, *p* = 0.01). Participants with higher stress variability exhibited a higher performance gap between target sizes, leading to the acceptance of H3a.

Shooting self-efficacy was negatively associated with stress variability (*r* = −0.18, *p* = 0.03), suggesting that participants with higher self-efficacy maintained more stable emotional states across the task, so H3b could be accepted.

## 5 Discussion

The present study examined the influence of target size, self-efficacy, stress, and shooting experience on precision in a military sample. Results showed that a smaller target area, while maintaining an identical target point, was associated with significantly higher shooting precision, as measured by MDC and shot group radius. This difference was not explained by subjective stress, suggesting that peripheral cues from the target area influence psychomotoric processes rather than altering stress response.

From an attentional perspective, the peripheral visual characteristic of the target area may act as perceptual anchor that influence not only subjective target size estimates but also attentional allocation and motor precision (Wesp et al., 2004; Witt et al., 2008). This supports the notion that, even with a constant target, changes in the perceptual context can influence visuomotor processes. The role of perception on psycho-coordinative task execution has been researched intensively in the realm of quiet-eye. Quiet eye as longer final fixations prior to movement initiation, has been shown to enhance fine motor adjustments during the terminal phase of movement execution (Vickers, 2007; Mann et al., 2007). Longer quiet eye durations are associated with higher performance levels, greater aiming accuracy, and are considered a critical period for movement programming in the aiming response (Vickers, 1996; Williams et al., 2002). Studies further indicate that elite shooters demonstrated an earlier onset and a longer relative duration of quiet eye, and a slower movement of the gun barrel than semi-elite shooters (Causer et al., 2010). In the present context, although task difficulty was objectively similar between both target conditions (medium-sized blue cross), the subjectively perceived difficulty of the smaller target area may have prolonged quiet eye duration, consistent with evidence that more challenging aiming tasks elicit longer final fixations (Williams et al., 2002).

Beyond gaze behavior, sensorimotor processes may also explain this effect. In particular, during the limb-target control phase, sensory feedback is used to spatially align the weapon with the target (Elliott et al., 2017). In this phase, shooters make continuous micro-adjustments to minimize deviation and maintain high-frequency alignment with the target point. Marković et al. (2020) demonstrated in their study that, particularly within one hundredth of a second before shot release, rotational speed (*r* = 0.41, *p* < 0.05) and weapon kinematics (*r* = 0.40, *p* < 0.05) influenced shooting precision. The peripheral cue of the target area could implicitly serve as an auxiliary reference, influencing weapon kinematics and the perceptual-motor alignment process. A smaller area may signal deviations from the aim point faster, triggering earlier corrective movements, whereas a larger area provides less immediate visual feedback about limb-target distance changes.

Taken together, the findings suggest that the performance advantage of smaller target areas likely arises from a combination of high-resolution visual attention, more precise proprioceptive alignment, and more precise weapon kinematics, rather than from altered subjective stress experiences. Even when the aim point remains constant, the surrounding target area appears to modulate the limb-target control phase by providing higher-resolution spatial reference information. This highlights the importance of considering both central and peripheral visual information in the design of training protocols aimed at optimizing shooting precision.

In addition to target size, psychological factors such as shooting self-efficacy and shooting experience also emerged as predictors of precision. Self-efficacy, as the beliefs in one’s capabilities to execute courses of action required to produce given attainments (Bandura, 1977) in the domain of shooting influenced performance for both the small and large target conditions. Moreover, shooting self-efficacy was negatively associated with subjective stress at all three measurement points, as well as with stress variability between these measurements. This supports the notion that confidence in one’s own competence facilitates goal-directed execution and task-relevant attentional control (Bédard Thom et al., 2021; Themanson and Rosen, 2015). Therefore, self-efficacy enhance performance through greater attentional allocation to task-relevant processes, higher resistance to distraction, and more consistent technical execution (Domínguez-González et al., 2024).

The effect of self-efficacy on shooting performance found in this study corresponds to the moderate relationship (*r* = 0.38, *p* < 0.001) reported in the meta-analysis by Moritz et al. (2000) across different sports disciplines. However, the present results only partially explain the mechanism through which self-efficacy affects precision. Subjective stress mediated the effect partially (17.7%), but the large proportion of variance on the effect between self-efficacy and precision remained unexplained. One possible explanation for this relationship could be that participants with higher self-efficacy were in fact more skilled marksmen due to greater training experience. Nevertheless, the relationship between shooting self-efficacy and precision remained nearly unchanged after controlling for shooting experience (big target: *r* = 0.36, *p* < 0.001; small target: *r* = 0.23, *p* = 0.006). Furthermore, Biggs et al. (2024) found that in a military sample, self-reports did not predict actual marksmanship abilities. This makes it unlikely that the observed self-efficacy effect is a spurious correlation driven by the relationship between shooting ability and precision.

The mechanism underlying the influence of shooting self-efficacy may therefore be predominantly psychological. High self-efficacy is generally associated with greater decisiveness in action and increased approach motivation in athletes (Rogowska et al., 2022). Approach motivation describes the tendency to move toward positive stimuli or goals (Elliot and Thrash, 2002) and to focus attention on goal-relevant cues (Gable and Harmon-Jones, 2010). In the present context, this may translate into an offensive and more stable body posture as well as more goal-oriented gaze behavior (see also quiet-eye literature). In contrast, low self-efficacy is often linked to avoidance motivation (Heimerdinger and Hinsz, 2008), in which the aim is not necessarily to achieve maximum precision but rather to avoid inaccuracy. Such an error-prevention focus could influence gaze behavior and limb–target control, potentially resulting in greater deviations. Ultimately, shooting self-efficacy may translate into performance differences through associations with approach or avoidance tendencies that influence movement characteristics such as gaze patterns and body positioning. Higher shooting self-efficacy and approach motivation could enhance, as Eberspächer (2024) summarizes, synchrony between thought and movement as a prerequisite for high precision in motor execution.

In addition to self-efficacy, subjective stress emerged as a factor influencing shooting precision, consistent with the findings of the meta-analysis by Cooper et al. (2024) on the relationship between pressure and marksmanship performance. The negative impact of subjective stress on precision can also be traced to its effects on gaze behavior and motor execution. In the study by Wilson et al. (2009), anxiety impaired visual attentional control by shortening quiet-eye duration and destabilizing gaze behavior. Similarly, Nieuwenhuys and Oudejans (2010) reported that, under high-anxiety conditions, shooting precision decreased due to faster trigger actions and a higher blink rate. Perceived stress therefore appears to influence attentional control, task execution, and, consequently, marksmanship performance (Guo et al., 2025).

Interestingly, the physiological stress measure of heart rate variability (RMSSD) in the present study showed no significant association with subjective stress, self-efficacy, or shooting precision. This finding contrasts with the results reported by Ortega and Wang (2018), who identified HRV as a predictor of both self-efficacy and marksmanship performance. Similarly, Thompson et al. (2015) found that the change in RMSSD between rest and shooting was a significant predictor of shooting time; however, RMSSD was not used as a predictor of precision in their analysis. In their study, more precise shooters exhibited smaller changes in autonomic responses during the task, as measured by low frequency power (LF) and total power (TP). Further, the LF/HF-ratio, interpreted as an indicator of sympathovagal balance, was shown by Zhuang et al. (2008) to be a valid performance-related measure and may therefore also be considered in future marksmanship research. Moreover, factors such as time-of-day effects (Medeiros et al., 2021) and sleep quality (Rauter et al., 2025) could have exerted unsystematic influences on RMSSD values in the present study and should be controlled for in future studies.

Finally, stress state variability, operationalized as the mean squared successive differences (MSSD) across the three measurement points, was examined. Based on the findings of Kayihan et al. (2013), who reported that anxiety variability was negatively associated with shooting precision in police officers, it was hypothesized that stress state variability would also be negatively related to performance stability across target conditions. The results revealed a positive association between stress state variability and performance stability, suggesting that lower emotional reactivity is linked to more consistent performance. Athletes with higher emotional stability tend to achieve better results, as they can manage stress effectively and maintain attentional focus (Kostikova and Romanina, 2014). Accordingly, interventions aimed at enhancing emotion regulation skills, such as mindfulness training, have proven effective in improving performance in competitive settings (Tingaz, 2025). Consistent with expectations, self-efficacy showed a negative association with stress state variability, in line with the notion that greater confidence in one’s abilities serves as an emotional stability factor (Doménech et al., 2024; Schmitt, 2008). Nevertheless, the positive relationship between stress state variability and performance stability should be further investigated, particularly under more dynamic operational conditions and stronger stressors, such as in force-on-force scenarios.

### 5.1 Limitations

This study provides new insights into how target characteristics, psychological dispositions, and physiological stress interact to shape shooting precision; however, several factors limit the generalizability of the findings. The sample consisted exclusively of officer cadets, predominantly young, active-duty soldiers of the German Armed Forces. Future research should include combat troops and units with varying specializations to improve representativeness. Additionally, shooting took place in a simulator that replicated realistic recoil but did not involve actual projectile discharge. While this setup enables experimental control, it may induce lower arousal than live-fire situations, potentially underestimating the influence of physiological and subjective stress responses. The controlled indoor environment further limits ecological validity, as it does not capture the sensory complexity and unpredictability of real operational contexts. Moreover, the fixed order of target presentation may have introduced order effects such as learning, adaptation, or fatigue, which could have contributed to performance differences between target sizes. Although the within-subject design controls for interindividual variability, it does not rule out that changes in performance were partly attributable to presentation order rather than target size per se. Finally, due to the absence of a validated German-language measure of shooting self-efficacy with the P8 pistol, a custom instrument was developed, which showed very good internal consistency but should be validated in future studies. Subsequent research should incorporate additional performance-related variables to better understand determinants of shooting precision, including handgrip strength and pistol movement via kinematic sensors (Marković et al., 2020), gaze behavior via eye-tracking (Williams et al., 2002), video analysis of body positioning (Choi et al., 2006), and fine-grained temporal measures to differentiate the impulse control phase from the limb–target control phase. Another methodological consideration concerns individual differences in shooting technique. Participants were not instructed to adopt a standardized stance or a specific one-eye versus two-eye aiming strategy, but were asked to shoot in their usual, comfortable manner. This approach maintains ecological validity, as operational shooting styles vary among trained personnel, but it also introduces uncontrolled variation in visual alignment and sight acquisition. For instance, eye dominance and handedness may influence how easily shooters acquire the target, particularly because the small target area was positioned on the upper left portion of the larger silhouette. Left-eye or left-hand dominant shooters may therefore have had a slightly different visual alignment advantage or disadvantage. Future studies should examine whether stance, eye dominance, and sighting strategy systematically affect precision under different target configurations and whether standardization or measurement of these factors can improve experimental control.

## 6 Conclusion

In conclusion, this study demonstrates that target size, shooting self-efficacy, subjective stress, and shooting experience all contribute to variations in marksmanship precision in a military context. Smaller target areas improved precision independent of subjective stress, likely via enhanced perceptual-motor alignment during the limb–target control phase. Self-efficacy emerged as a robust psychological predictor, partly mediated by subjective stress, whereas physiological stress indices such as RMSSD were unrelated to precision. Further, stress state variability was positively associated with performance stability, indicating emotional regulation trainings like mindfulness as effective for performance enhancement in high-risk occupations. These findings highlight the multifactorial nature of marksmanship and underline the need to consider perceptual, psychological, and physiological factors jointly in both research and applied training design.

## Data Availability

The datasets presented in this study can be found in online repositories. The names of the repository/repositories and accession number(s) can be found at: https://osf.io/w72pn/.

## Footnotes

1
https://osf.io/z2df5
